# Human-nature relationships in context. Experiential, psychological, and contextual dimensions that shape children’s desire to protect nature

**DOI:** 10.1371/journal.pone.0225951

**Published:** 2019-12-05

**Authors:** Matteo Giusti

**Affiliations:** Department of Building Engineering, Energy Systems and Sustainability Science, University of Gävle, Gävle, Sweden; Università degli Studi di Perugia, ITALY

## Abstract

What relationship with nature shapes children’s desire to protect the environment? This study crosses conventional disciplinary boundaries to explore this question. I use qualitative and quantitative methods to analyse experiential, psychological, and contextual dimensions of Human-Nature Connection (HNC) before and after children participate in a project of nature conservation. The results from the interviews (N = 25) suggest that experiential aspects of saving animals enhance children’s appreciation and understanding for animals, nature, and nature conservation. However, the analysis of children’s psychological HNC (N = 158) shows no statistical difference before and after children participate in the project. Analysing the third dimension–children’s contextual HNC–provides further insights. Including children’s contextual relations with home, nature, and city, not only improves the prediction of their desire to work for nature, but also exposes a form of Human-Nature Disconnection (HND) shaped by children’s closeness to cities that negatively influence it. Overall, combining experiential, psychological, and contextual dimensions of HNC provides rich insights to advance the conceptualisation and assessment of human-nature relationships. People’s relationship with nature is better conceived and analysed as systems of relations between mind, body, culture, and environment, which progress through complex dynamics. Future assessments of HNC and HND would benefit from short-term qualitative and long-term quantitative evaluations that explicitly acknowledge their spatial and cultural contexts. This approach would offer novel and valuable insights to promote the psychological and social determinants of resilient sustainable society.

## Introduction

Living within sustainable boundaries is a challenge that concerns this and the future generations of humans [1]. How future generations will develop the desire to protect the environment and live sustainably is a research subject that is receiving exponential attention [2]. Across environmental and conservation psychology [3,4], landscape management [5,6], biological conservation [7,8] and social-ecological sustainability research [9–12], a personal connection with nature is considered a core determinant for environmental protection and sustainable living. However, many in academia recognise that the ability to appreciate, and eventually protect, the biosphere is threatened by children’s lack of direct nature experiences [13–16] and by the increasing virtualisation of children’s lives [17–19]. These pressures—and the urgent need to create sustainable living standards—are driving a new multidisciplinary arena that investigates how psychological and social determinants of sustainable societies develop in people [4,20–22]. Hence, human-nature relationships are studied across many disciplines, but oftentimes disciplinary boundaries limit the valuable integration of the complementary insights produced [2]. This study addresses this interdisciplinary research gap.

This study aims to advance the conceptualisation and assessment of human-nature relationships to better understand what promotes children’s desire to protect nature. To achieve this aim, this study investigates how participating in a project of nature conservation affects children’s human-nature relationship. In the sections below, I introduce the different dimensions of human-nature relationships that exist within disciplines, I described how the study is designed and the conservation project under examination, and then list the methods used. Afterwards, I describe the results of the study, summarise them in the context of the nature conservation project, and then discuss how they contribute to improving the conceptualisation and assessment of human-nature relationships to better predict children’s desire to work for nature in the future.

### Psychological, experiential, and contextual human-nature connection

Human-Nature Connection (HNC) is a concept that emerges from a multidisciplinary review of the body of knowledge on human-nature relationships [2]. This concept joins three complementary dimensions of human-nature relationships that are often studied in isolation from each other. The first dimension (*psychological HNC*) emerges from research that considers human-nature relationships as an attribute of the mind. This body of literature studies the psychological connection to an abstract form of nature. Changes in people’s connection with nature are measured using quantitative methods often to describe psychological dynamics or to predict specific pro-environmental behaviours (for examples see [23,24]). The second dimension (*experiential HNC*) is representative of qualitative research that describes human-nature relationships as experiences of being in nature. Here, researchers observe and describe people’s interaction with local nature (for example see [25]). The last dimension (*contextual HNC*) emerges from research on ‘sense of place’ and it investigates human-nature relationships as the sense of belonging that people develop through time with geographical areas. Typically, these studies use questionnaires to study people’s attachment to specific natural landscapes (for review see [6]).

Despite these psychological, experiential, and contextual dimensions of human-nature relationships being investigated and reported on, single studies usually focus only on one dimension. In doing so, the valuable cross-fertilization across these bodies of knowledge is largely missing [2]. Beyond the missed opportunity for valuable interdisciplinary insights, disciplinary boundaries have shown to limit the analysis of human-nature relationships. For instance, the predictive power of psychological HNC alone for pro-environmental behaviours is limited when contextual factors are introduced [26–28]. Duffy and Verges [26] show that seasonal and meteorological factors meaningfully influence people’s association with nature. Contextual influences to psychological HNC are also evident when the RSPB [29] reports that, somehow counterintuitively, British children are psychologically closer to nature in urban rather than in rural areas. Geographical access to nature experiences is shown to promote children’s psychological HNC [30], but it stands to question to what kind of nature children develop their appreciation for. Not all nature experiences are equal [31–33] and there is initial indication that different kinds of nature experiences contribute to different aspects of children’s relationship with nature [34]. This study operationalises, analyses, and discusses the three dimensions of HNC jointly and offers interdisciplinary insights to address some of these limitations.

### Study design

Empirically, this study focuses on children. This is because nature experiences during childhood can promote the psychological foundation for a multitude of environmentally conscious behaviours [35–37] and for an adult life devoted to environmental protection [38–41]. In this study, the *experiential* dimension of HNC is assessed qualitatively after children participate in a project of nature conservation (see section below for details). The impact of this nature experience on children’s *psychological* and *contextual* dimensions of HNC is then quantitatively evaluated. This numerical data is analysed to understand what best predicts children’s desire to protect the environment in the future or work for environmental organisations.

The design of this study responds to the need to analyse all dimensions of HNC simultaneously, by using a multi-method approach on a large dataset of participants in combination with some control over socio-demographic factors [42]. This design addresses two critiques common to retrospective research on nature experiences: first, participants have a nearly identical socio-cultural background (e.g. age, level of education, and culture) and, second, they are assessed when memories are still vivid [43]. This multi-dimensional and interdisciplinary investigation provides a set of results useful to discuss the constituents of human-nature relationships and to debate what shapes children’s desire to protect nature.

### The salamander project

The Salamander Project (SP) is a voluntary program of nature conservation involving 10-year-old students who attend a municipal school in the outskirts of Stockholm (Sweden). These students are responsible for saving and documenting two endangered species of salamanders. During every school day from April to May, a group of 5 to 8 children walk to a local park guided by a schoolteacher. Every time the group of children is likely to be different. In the park, there is a dry paddling pool in which salamanders frequently fall in to on their way to a nearby breeding ground, remain trapped, dry, and consequently die. So, participating in the SP means that children go into the dry pool, look for salamanders, pick them up out of the pool by hand, document species and gender, and then release the salamanders into the nearby pond where they can reproduce. All children from the 4^th^ grade in this school are invited to participate, but participation is voluntary. Still, the SP is a flagship of pride for this school and children usually participate happily. Overall, the SP is an authentic project of nature conservation [44] with documented ecological success [45,46] and it is not conceived by the school as a pedagogical activity. Nonetheless, the SP has inherent educational value for children as Barthel et al. [44] have documented.

## Methods

### Participants

Participants are 158 (85 males, 73 females) 10-year-old students attending three schools in Stockholm. Only one school takes part in the SP. The two schools not partaking in the SP act as control groups to ensure a balanced number of subjects between control and treatment groups. These schools are within 3 kilometres of each other to maintain social-economic factors and spatial access to natural environments outside the SP nearly constant. All schools are recruited via personal contacts and phone calls and they decide to participate in the study to know more about their pupils’ relationship with nature.

Participants attending one school (N = 67, 3 classes) partake in the SP, whereas the students attending the other two schools do not participate (N = 91, 4 classes). Of the 67 children partaking in the SP, 25 children are interviewed after participating in the SP. This group of children is recruited on voluntary basis. However, the final selection is made to ensure that this group is equally distributed across the three classes involved in the SP, that there is equal gender representation (12 males, 13 females), and that there is variety in the number of times children directly participate in collecting salamander (2 to 5 times).

### Materials

#### Experiential HNC

Experiential HNC is assessed by interviewing children after they participate in the SP. The interviews follow an interview guide that focuses on what children think and feel about the SP, salamanders, animals, and nature in general (S1 Appendix).

#### Psychological HNC

In order to evaluate different, and potentially complementary, aspects of psychological HNC, I assess both ‘connection to nature’ [47] and ‘connectedness with nature’ in its explicit (i.e. available to consciousness) and implicit (i.e. outside of conscious awareness) form [48,49]. These concepts refer to independent methods to assess psychological HNC. Connection to nature is assessed using the Connection to Nature Index (CNI) from Cheng and Monroe [47]. This is known to be a reliable tool to use with 10-year-old children alike the participants in the study [50–53]. In addition, I revise four items from the CNI subscale ‘empathy for creatures’ to assess exclusively empathy for salamanders (hereinafter called *salamander empathy*) (S2 and S3 Appendix). The scale salamander empathy is created to capture potential emotional changes specifically towards salamanders since the SP focuses on saving these animals.

**Fig 1:**
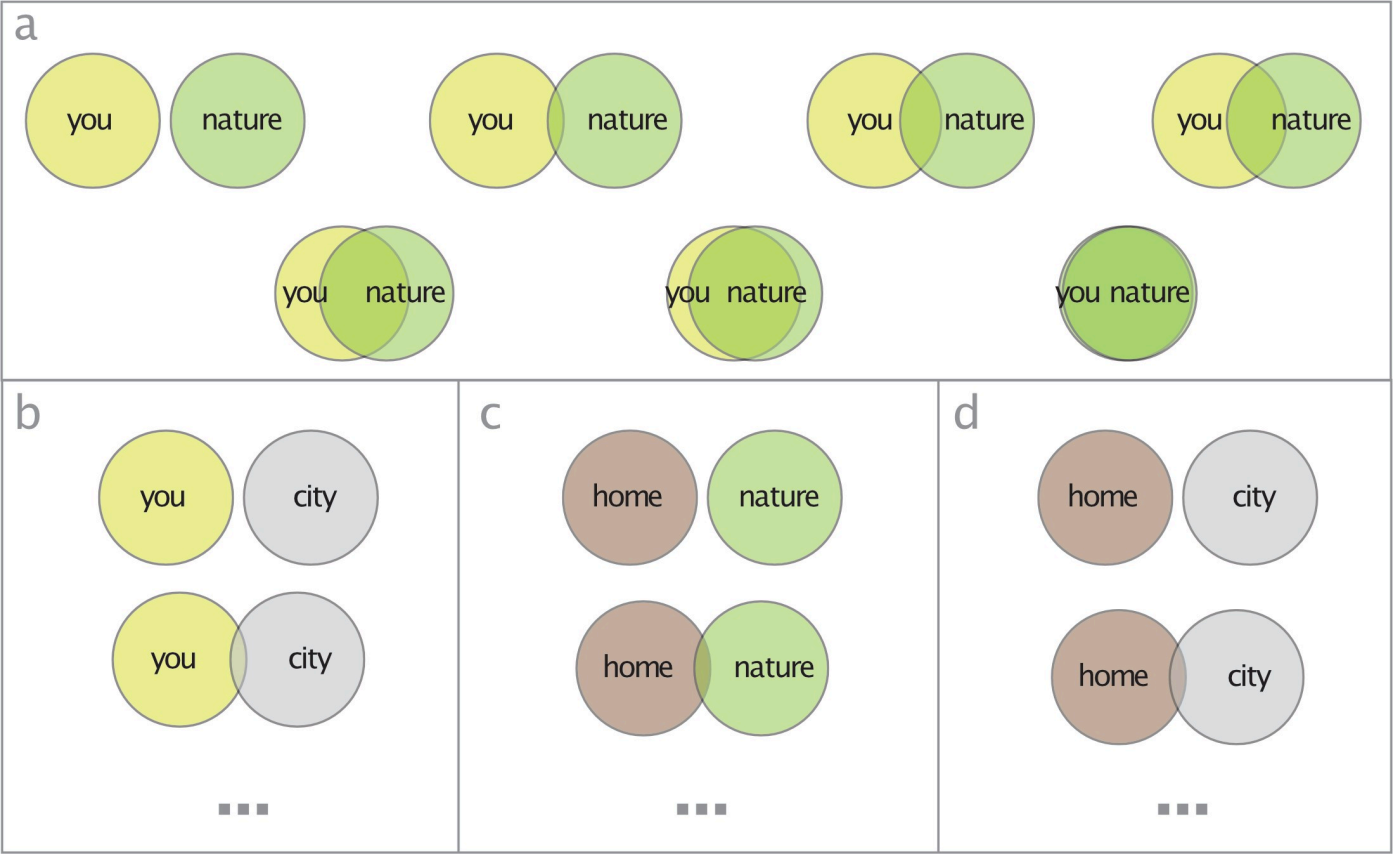
Diagrams used for analysis of explicit connectedness with nature and contextual HNC. The four diagrams used to assess children’s: self-nature closeness (i.e. INS) (a), self-city closeness (b), home-nature closeness (c), home-city closeness (d).

Explicit connectedness with nature is assessed using the Inclusion of Nature in Self scale (INS) [49], because its graphical form (Fig 1A) is easy to understand for children [53–55] and because it reports consistent results over time [56]. This method can also be considered an assessment of children’s self-nature closeness [57]. Implicit connectedness with nature is assessed using a computerized Implicit Association Test called FlexiTwins [48]. This is a videogame that measures how quickly children can associate with words representing the built environment or representing nature. How much faster children’s reaction times are with words representing nature is an indication of their implicit association with nature. FlexiTwins is chosen because it can reduce biased results, given that children might be incapable to fully articulate their association with nature [56,58,59]. FlexiTwins is already used in studies with young people [48,60].

Lastly, after the treatment group participated in the SP, all participating children answer in written form to the question: “Would you like to work to protect nature or in environmental projects in the future?”. All the above material is translated to Swedish to make it accessible to Swedish children (see S2 and S3 Appendix).

#### Contextual HNC

The analysis of the contextual dimension of HNC is based on understanding relations among four concepts: *self*, *nature*, *home*, and *city*. In other words, it means understanding how children perceive themselves, and their homes, integrated in the concepts of nature and city. Home is unanimously considered the place to which people attach the most meaning [61]. Home is the context people use as a reference point to construct reality in their daily life [62]. In an extensive review of sense of place literature, Lewicka [6] writes: “Home is a symbol of continuity and order, rootedness, self-identity, attachment, privacy, comfort, security and refuge”. How nature is integrated in the concept of home, is a contextual relation worth exploring to understand how contextual HNC influence children’s desire to work for nature.

Another central concept for contextual HNC is city. Cities are amongst humankind’s greatest inventions and amongst the biggest challenges to sustainability [63]. They are worth names and they are elements of pride and conflicts in the history of humankind [6,64]. However, they have the peculiarity of being environments exclusively constructed for human use, from which natural dynamics are mostly separated and hidden [65]. Thus, how children perceive themselves and their homes in relation to the concept of city is crucial to unveil how their attachment to this context might shape their relationship with nature and influence their desire to work for nature.

The contextual dimensions of HNC are analysed with three variants of the Inclusion of Other in the Self scale [57] and three sets of questions. First, I use seven pairs of increasingly overlapping circles to quantify children’s self-city closeness (Fig 1B), home-nature closeness (Fig 1C), and home-city closeness (Fig 1D). This scale is the same as the one used to assess self-nature closeness, or INS, in the assessment of psychological HNC. Additionally, children are asked to answer in written form to three questions for each context selected (i.e. *city*, *home*, *nature*): “what does [city/home/nature] mean to you?”, “what is best about [city/home/nature]?”, and “what is worst about [city/home/nature]?”. These questions unpack the mental representations that children use when they are asked about their closeness to nature and city, and their home-nature and home-city closeness. Unfolding the attributes that constitutes children’s *home*, *nature*, and *city* allows investigating the set of meanings that form the foundation of children’s contextual HNC. All children’s written answers are coded using a thematic analysis. All the material above is translated to Swedish to make it accessible to Swedish children (see S2 and S3 Appendix).

### Procedure

#### Experiential HNC

Children’s experiential HNC is assessed through interviews within one week from participating in the SP (June 2015). All interviews are held in Swedish at the school during school time and they last about 10 minutes each. They are recorded, transcribed, and inductively coded using Atlas.ti following the systematic process described by Braun and Clarke [66].

#### Psychological and contextual HNC

Children’s psychological and contextual HNC are assessed at the school that children attend during school hours using printed material. Participants receive brief oral instructions about the content of the activity and then are provided with a printed booklet for the assessment (S3 Appendix). All children are given sufficient time to complete the whole assessment in a reasonably distraction-free environment. After completing all written questions in the booklet, children are asked to move to a computer lab, or use laptops, to play an offline version of FlexiTwins. This assessment is performed on all children twice: before some of them participate in the SP (April 2015) and after (June 2015).

### Ethical protocol

The ethical protocol of this study has been approved by the responsible ethical committee (Stockholm Resilience Centre, Stockholm University). The author had a background police check which was provided to all schools before fieldwork. All participants’ parents or guardians received an informative letter about the study and provided written consent to allow children to participate in the study. This consent allowed the author to record the interviews, analyse results, and utilise quotes from the interviews anonymously. The names reported in the quotes are fictional.

## Results

### Experiential HNC

The results from interviews suggest that (i) all 25 children interviewed perceive salamanders in a different light after participating in the SP. In the eyes of these children, salamanders are now associated with aesthetic pleasure, feelings of care, empathy, and respect. Children wonder where salamanders are at night, how they feel, and what kind of life they have. In other words, children show the capacity to imagine what the life of a salamander is, and show concern for their livelihoods. The quotes below represent this finding.

“I have started to like them and I know now that you have to be careful with them.” (Sky)

“I had seen a salamander before (…) but I didn’t know so much about them. And now it feels like ‘Oh, I want to have my own salamander’! They are so smooth and soft! (…) They are so nice!” (Ellie)

“First I felt, well … how can I explain? ‘Yeah it’s exciting but they are …like …just salamanders’. But now I feel more like, they are alive, they exist. Before I didn’t think ‘I wonder where they are.’ […] I care more about them.” (Loreta)

“I have more […] respect for how they live because it’s quite … I wouldn’t survive if I were a salamander! … Now I see them in a different way. Before I thought they were like animals. Now it’s like they are beings that, well, they need help, just like people can need help sometimes.” (Roberto)

These changes are not limited to children’s relation to salamanders. By immediate generalization, children transpose these insights to others animals. Most children state that participating in the SP has altered their relations to animals and nature in general, as the quotes below show.

“I have started to think more about animals and nature. Actually a lot more than what I did before.” (Ale)

“That you shouldn’t harm them because they are also animals and they often live a hard life.” (Quentin)

“Yes, well, I have much more of a sense for nature.” (Megan)

“Well, it’s like I’m less scared and I feel … more confident in nature.” (Stephan)

Notably, children’s narratives repeatedly report a specific formative moment. The emotional reaction linked to finding salamanders is often associated with overcoming, what I call here, the ‘yuck barrier’ (ii). Once a child finds a salamander, it is his or her responsibility to collect it by hand and determine species and gender. Many children say that before participating in the SP they thought that holding a salamander is simply ‘yuck’, and this emotional threshold kept them from touching, or even considering touching, these animals. However, participating in the SP puts them in a position of responsibility that forces them to overcome this emotional barrier, as the quotes below show.

“They were like this little slimy, I didn’t dare to hold it, but now I feel like, now I can hold one without a problem.” (Liz)

“The first time it was a little scary. I'm a little afraid of animals so then it was a bit scary, if it would bite me or how it would feel if it was on my hands. It was a little nerve-racking the first time. […] Then it was completely normal.” (Filippo)

All children are enthusiastic about participating in the SP. Moreover, the first-hand experience of saving the life of a salamander makes them understand the moral rationale that underpins nature conservation, as the quote below shows.

“I have learnt to take care of animals. I'm maybe thinking about doing something like that maybe …to fix things so that everything is good with nature. (…) Yes …I have become more nature-friendly.” (Johan)

Collectively, these results show that participating in the SP changes children’s relation to salamanders, other animals, and nature in general. The importance of the SP in shaping children’s relationship with nature is also confirmed in a follow-up study with the same children two years after they participated in the SP [44].

### Psychological and contextual HNC

The statistical analyses of psychological and contextual HNC are presented together because they share the same quantitative methodology and because they provide similar insights.

#### Data screening

All data from FlexiTwins have to be screened for consistency following a standard procedure prior to analysis (for details see [60]). Consequently, all participants’ results that have error rates above 65% (n = 21) or that have inconsistent scores within the two phases of the same session (n = 27) are deleted. Overall, 48 of the 158 participants are excluded from the analysis. This is a representative outcome for 10-year-old participants outside laboratory conditions. Children’s excitement of playing a videogame and comparing their performances undermine the focus that FlexiTwins requires to obtain accurate results.

#### Data overview

The software R [67] is used to perform ANOVA tests and independent t-tests on the psychological and contextual dimensions of HNC. The data are reported with p-values between 0.1 and 0.9 as nonsignificant, whereas other results are interpreted according to their p-values, effect size, and in terms of scientific importance and relevance [68,69]. Table 1 shows an overview of the resulting data. The main results are presented separately in the following sections.

**Table 1:** Results of t-test analysis (means, t, df, p-values, effect size d) for differences in psychological and contextual HNC due to baseline, due to impact of the SP, and due to gender.

	*baseline*	*impact of SP*	*gender*
	*(control-treatment)*	*(pre-post)*	*(male-female)*
Psychological HNC			
*FlexiTwins*	M_C_ = 0.66±0.12	M_PRE_ = 0.59±0.15	M_M_ = 0.60±0.12
	M_T_ = 0.61±0.12	M_POST_ = 0.59±0.14	M_F_ = 0.65±0.12
	t(99) = 1.96 p = .053	p>.1	t(212) = 2.84 p < .01
	d = .39		d = .39
*salamander*	M_C_ = 0.63±0.25	M_PRE_ = 0.79±0.18	M_M_ = 0.68±0.22
*empathy*	M_T_ = 0.76±0.21	M_POST_ = 0.81±0.19	M_F_ = 0.78±0.19
	t(136) = 3.22 p < .01	p>.1	t(279) = 3.86 p < .001
	d = .55		d = .46
*CNI*	M_C_ = 0.71±0.16	M_PRE_ = 0.73±0.18	M_M_ = 0.67±0.17
	M_T_ = 0.73±0.18	M_POST_ = 0.74±0.15	M_F_ = 0.78±0.13
	p>.1	p>.1	t(280) = 5.81 p < .001
			d = .69
*self-nature*	M_C_ = 0.63±0.22	M_PRE_ = 0.63±0.22	M_M_ = 0.61±0.17
*closeness*	M_T_ = 0.64±0.22	M_POST_ = 0.65±0.19	M_F_ = 0.68±0.22
*(INS)*	p>.1	p>.1	t(279) = 2.87 p < .01
			d = .34
*desire to work*	NA	NA	M_M_ = 0.60±0.38
*for nature*			M_F_ = 0.81±0.29
			t(138) = 3.88 p < .001
			d = .66
Contextual HNC			
*home-nature*	M_C_ = 0.58±0.28	M_PRE_ = 0.62±0.25	M_M_ = 0.57±0.27
*closeness*	M_T_ = 0.63±0.25	M_POST_ = 0.59±0.21	M_F_ = 0.62±0.21
	p>.1	p>.1	t(274) = 1.76 p = .079
			d = .21
*self-city*	M_C_ = 0.56±0.25	M_PRE_ = 0.63±0.22	M_M_ = 0.59±0.25
*closeness*	M_T_ = 0.54±0.26	M_POST_ = 0.65±0.19	M_F_ = 0.54±0.23
	p>.1	p>.1	t(279) = 2.19 p = .029
			d = .26
*home-city*	M_C_ = 0.55±0.27	M_PRE_ = 0.48±0.27	M_M_ = 0.56±0.27
*closeness*	M_T_ = 0.48±0.27	M_POST_ = 0.53±0.24	M_F_ = 0.51±0.24
	p>.1	p>.1	t(276) = 1.68 p = .094
			d = .20

M_C_: mean control group; M_T_: mean treatment group; M_M_: mean male; M_F_: mean female; M_PRE_: mean group before SP; M_POST_: mean group after SP; NA: Not Available

p-values legend: 0.1>p>0.01: reported (light green)—p<0.01: green—p<0.001: dark green

#### Baseline differences

There are no baseline differences across schools or classes between control and treatment groups for any of the methods employed for contextual HNC (p>.1 for home-nature, self-city, and home-city closeness). There are also no baseline differences for the methods employed for psychological HNC (p>.1 for CNI and self-nature closeness), but for FlexiTwins (p = .053, d = .39) and salamander empathy (p = .002, d = .55). For FlexiTwins, that means that the *control* group has a higher implicit association with nature than the *treatment* group, but the difference is moderate and statistically weak. For salamander empathy, that means that empathy towards salamanders is higher in children who will participate but have not yet participated in the SP (iii). In this case, the difference is quite large and statistically strong.

#### Impact of participating in the Salamander Project

There are no statistical differences before and after children participated in the SP for any of the methods employed for psychological or contextual HNC (p>.1 for FlexiTwins, CNI, salamander empathy, self-nature, home-nature, self-city, and home-city closeness). Additional t-tests calculated on the mean difference between pre-SP and post-SP confirm these results. These results suggest that participating in the SP does not influence children’s psychological or contextual HNC (iv).

#### Gender differences

There are statistical differences due to gender in both sessions of assessment. Females show significantly higher psychological and contextual HNC than males (v). This is true for all the quantitative methods employed (.094<p < .001). The differences range from small for contextual HNC (0.20<d<0.26 for home-nature, self-city, and home-city closeness), to moderate for self-nature closeness (d = 0.34), FlexiTwins (d = 0.39), and salamander empathy (d = 0.46), and large for CNI (d = 0.69).

#### Meanings of *city*, *home*, and *nature*

**Fig 2:**
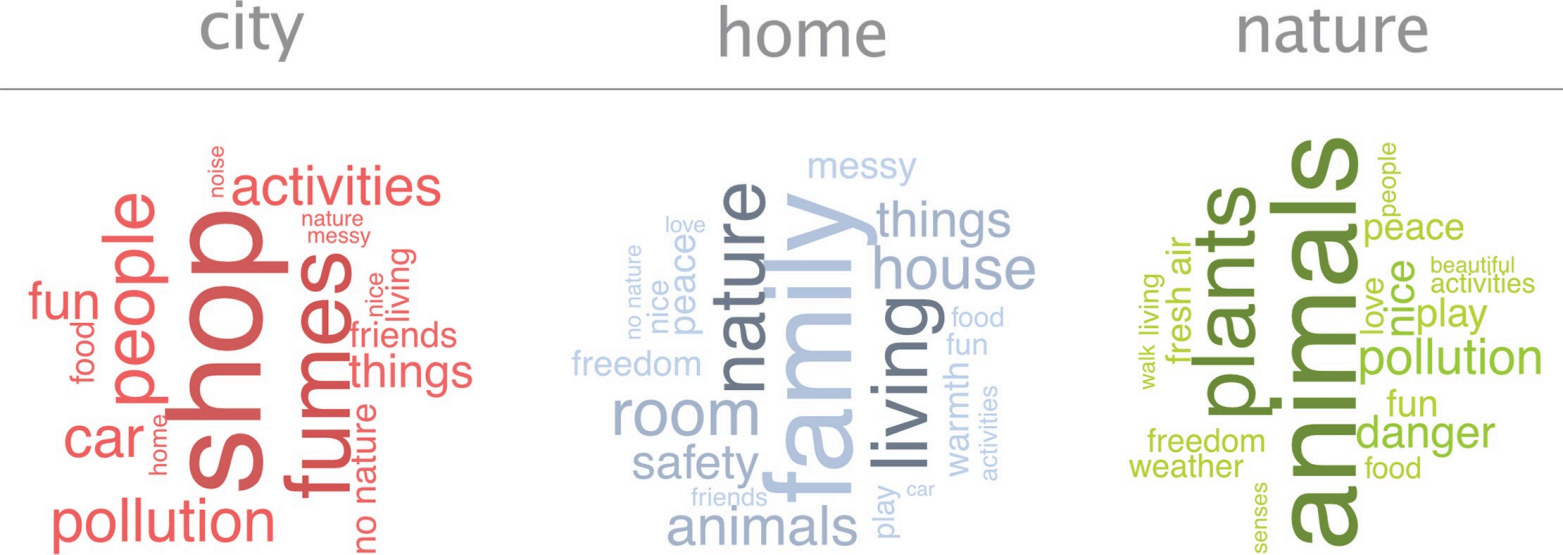
Word clouds of themes for city, home, and nature. The themes are reported more than three times. The size of the words is weighted for how many times the theme reoccurs in children’s answers.

The thematic analysis of how children describe the concepts of *city*, *home*, and *nature* produces 34 themes reported more than 1000 times (Fig 2). These themes are valuable insights into what children assume city, home, and nature to be when they are asked about their relations with such concepts in psychometric analysis. For instance, children do not conceive the concept of *city* in terms of geographical size, urban density, or trading opportunities, but the 10 most frequently reported themes are: shop (84), fumes (54), people (41), pollution (32), car (30) activities (28), fun (25), things (21), no nature (14), and friends (13). These are the themes the constitute the concept of city to which children are more or less close to. Differently, the 10 most frequent themes for *home* are: family (60), living (35), nature (34), room (28), house (25), animals (23), things (20), safety (19), messy (13), and peace (13). Lastly, the 10 most frequently reported themes that construct children’s concept of *nature* are: animals (94), plants (65), pollution (31), danger (26), nice (25), play (23), fun (21), peace (20), fresh air (19), freedom (16), and love (16). These results show that what children mean by city, home, and nature is a collection of meaningful everyday activities (e.g. shopping, living, playing), social contexts (e.g. people, family, friends), and emotions (e.g. fun, safety, peace) (vi).

### Predicting children’s desire to work for nature

**Fig 3:**
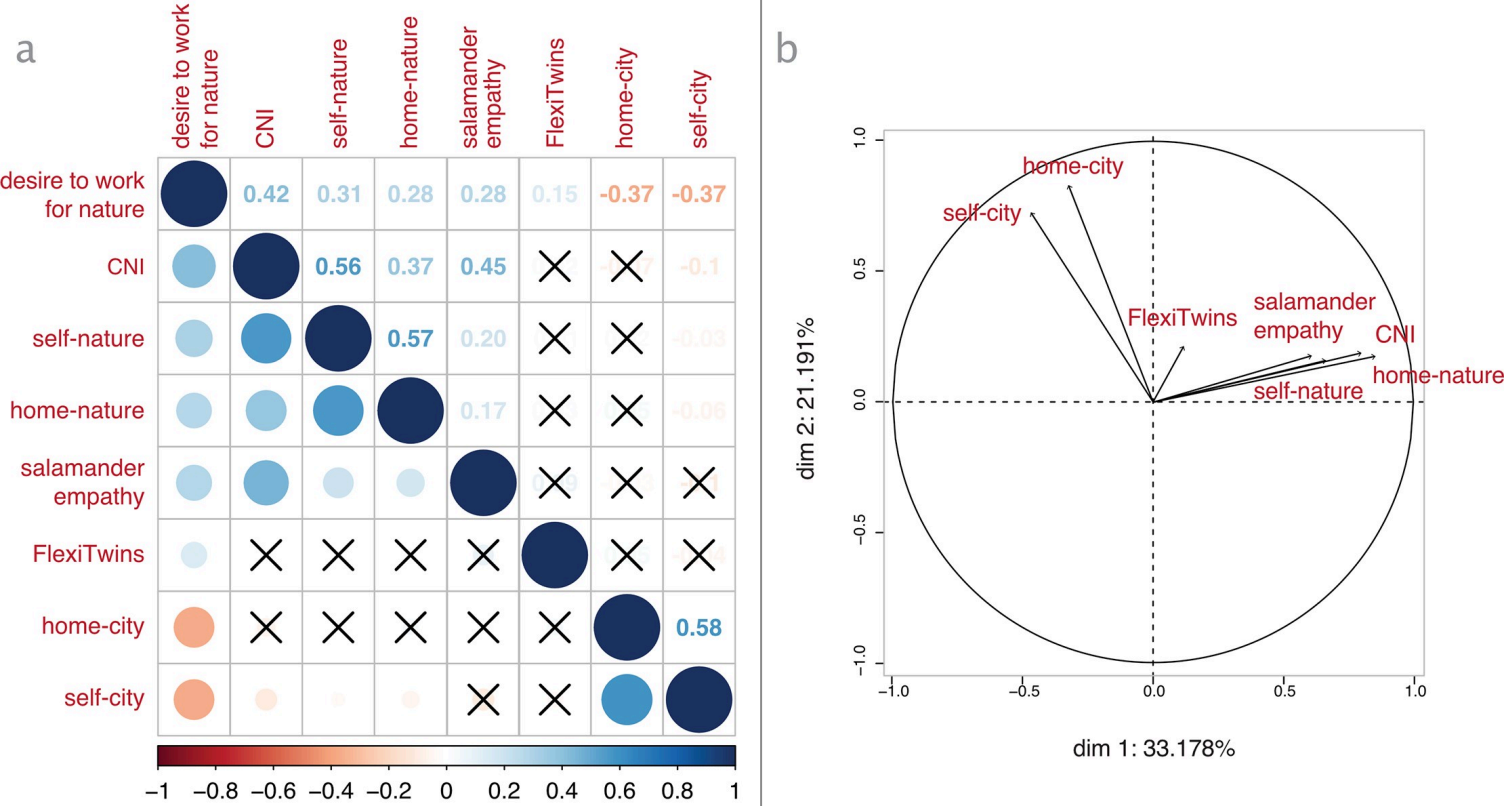
Correlation table and principal component analysis. (a) Spearman correlation table for all quantitative methods employed. Crossed elements are non-significant (p>.1). The strength of correlations is reported in the upper triangle. (b) Two-dimensional visualization of coordinates obtained from the principal component analysis for children’s desire to work for nature.

All children answer the question about their desire to work for nature by writing yes, no, or maybe (N-no = 19; N-maybe = 48; N-yes = 75). In line with all other methods, females show a higher desire to work for nature than males (p < .001, d = 0.66) (v). This difference is large and very significant. A correlation analysis (Fig 3A) shows that children’s desire to work for nature significantly correlates with CNI (p = 0.42), self-nature closeness (p = 0.31), home-nature closeness (p = 0.28), salamander empathy (p = 0.28), and FlexiTwins (p = 0.15), but it correlates *negatively* with home-city (p = -0.37), and self-city closeness (p = -0.37).

Bartlett’s sphericity test shows that the data are adequate for factor analysis (p<2.2e-16). The consequent principal component analysis shows that the majority of variance in this set of variables is explained by two opposing factors (Fig 3B and Table 2). In order to investigate the opposing drivers at play in how children learn the desire to work for nature, and because FlexiTwins, self-nature, and CNI are similar conceptualisations of one’s relationship with nature, I choose to further analyse two sets of composite variables. The first one, termed Human-Nature Connection (HNC), includes all variables that positively correlate to children’s desire to work for nature: CNI, self-nature closeness, home-nature closeness, salamander empathy, and FlexiTwins. The second one, termed Human-Nature Disconnection (HND), includes the two variables that negatively correlates to children’s desire to work for nature: self-city and home-city closeness.

**Table 2:** Results of principal component analysis.

*variable*	*M*	*SD*	*Factor 1*	*Factor 2*	*Factor 3*
Human-Nature Connection					
*CNI*	0.643	0.198	0.834	0.172	-0.030
*self-nature*	0.646	0.185	0.781	0.185	-0.250
*home-nature*	0.586	0.204	0.648	0.156	-0.202
*salamander empathy*	0.727	0.227	0.594	0.174	0.249
*FlexiTwins*	0.481	0.169	0.143	0.210	0.897
Human-Nature Disconnection					
*self-city*	0.587	0.227	-0.402	0.766	-0.174
*home-city*	0.554	0.231	-0.250	0.856	-0.018
% of variance			33.178	21.191	14.299

Means (M), standard deviations (SD), factor loadings, and % of variance explained.

**Fig 4:**
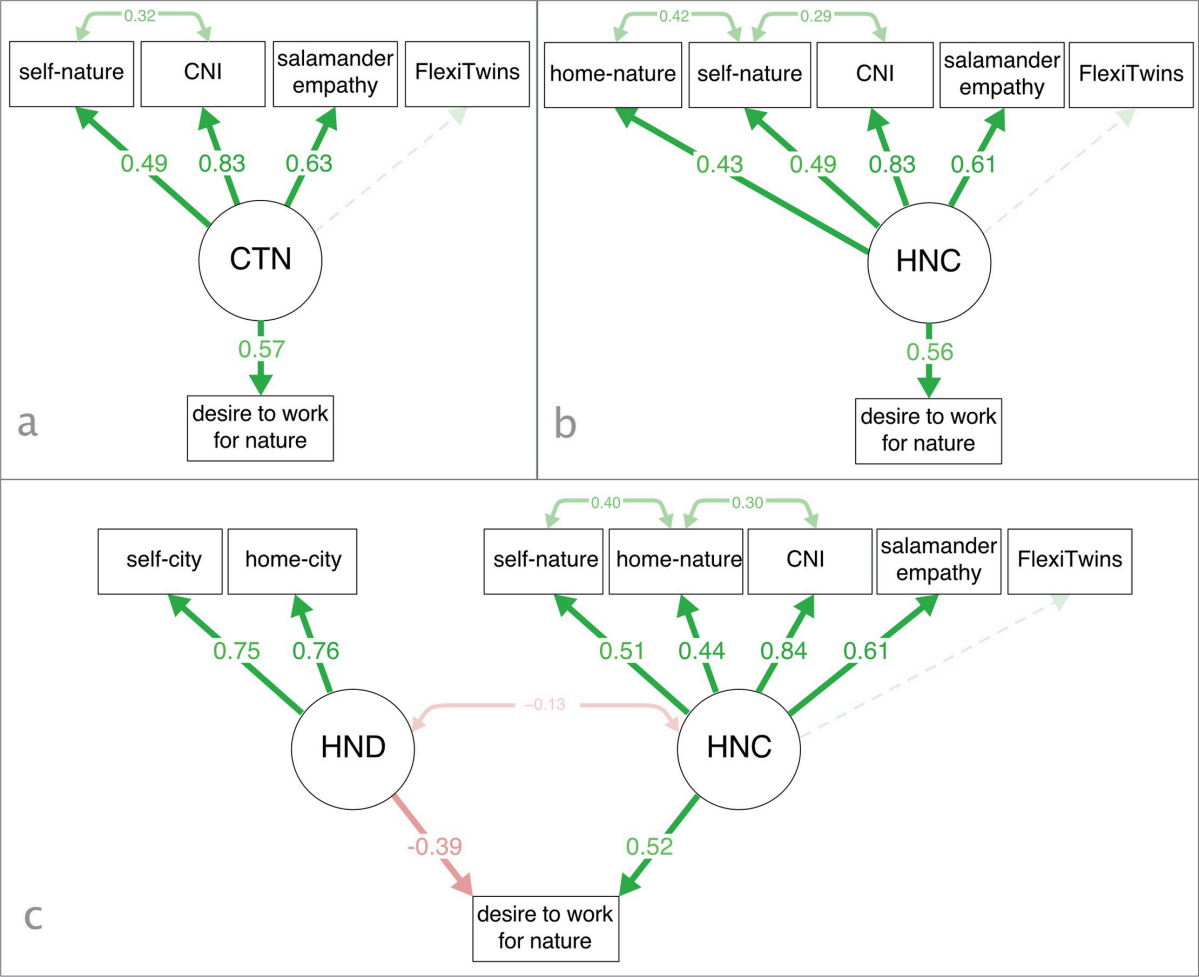
Three structural equation models to predict children’s desire to work for nature. a) CTN model; b) HNC model; c) HND-HNC model. Latent variables are in circles, measured variables in rectangles, and the lines show standardized parameter estimates.

The factors HND and HNC are treated as latent constructs in the structural equation model called *HND-HNC model* (Fig 4C). The HND-HNC model is compared to other three to appreciate which one offers the best fit for predicting children’s desire to work for nature (Fig 4). The first comparative model is the measurement model, which predicts children’s desire to work for nature by using only observed variables. The second comparative model (*CTN model*) introduces a latent construct based on psychological conceptualisations of people’s relationship with nature (Fig 4A). The third comparative model (*HNC model)* integrates positive contextual relations with children’s psychological HNC (Fig 4B).

Following the guidelines provided by Awang [70], I use multiple fit indices to evaluate the good fit of the models (p-value, CFI, RMSEA, GIF, chisq/df) and to compare them with each other (chisq/df, AIC, BIC). The analysis of fitness and comparison of all models is summarized in Table 3. The analysis of fitness shows that the measurement model does not fit the data (p>.5, CFI < .9, RMSEA>.08, chisq/df>3.0). The CTN model has almost acceptable fit to the data (RMSEA>.08), whereas both HNC and HND-HNC models have a good fit to the data. In all models, all standardized parameter coefficients, but FlexiTwins (.116<p< .199) are very significant (p < .001). The fit indices show that integrating home-nature closeness in the CTN model to contextualise children’s positive relationship with nature improves the fit of the data for all indices (see RMSEA, chisq/df, AIC, and BIC in Table 3 between CTN and HNC model) (vii). However, across all indices, the best model to predict children’s desire to work for nature is the HND-HNC model. In this model, self-city and home-city closeness are indicators of a relationship with nature that is negatively linked to children’s desire to work for nature (viii). Additionally, in the HND-HNC model the percentage of explained variance for children’s desire to work for nature passes from 32.7% (CTN model), and 31.7% (HNC model), to 47.2% (HND-HNC model).

**Table 3:** Fit indices for the four models studied.

	*models*			
	*measurem*.	*CTN*	*HNC*	*HND-HNC*
*Fitness values*				
*p-value*^*1*^	.000	.054	.195	.270
*CFI*^*2*^	.749	. 958	.982	.987
*GFI*^*3*^	.909	.972	.975	.965
*Df*	13	4	7	16
*RMSEA*^*4*^	.159	.102	.058	.039
*Comparison values*				
*chisq/df*	4.23	2.32	1.41	1.18
*AIC*		-202.0	-277.5	-354.4
*BIC*		-170.7	-237.9	298.1
*R*^*2*^ *desire to work*		32.7	31.7	47.2
*for nature*				
	*models*			
	*measurem*.	*CTN*	*HNC*	*HND-HNC*
*p-value* ^*1*^	.000	.054	.195	.270
*CFI* ^*2*^	.749	. 958	.982	.987
*GFI* ^*3*^	.909	.972	.975	.965
*RMSEA* ^*4*^	.159	.102	.058	.039
*Df*	13	4	7	16
*chisq/df* ^*5*^,^*6*^	4.23	2.32	1.41	1.18
*AIC* ^*6*^		-202.0	-277.5	-354.4
*BIC* ^*6*^		-170.7	-237.9	298.1
*R*^*2*^ *desire to work*		32.7	31.7	47.2
*for nature* ^*6*^				

Acceptable indices are highlighted in green, the darker the green the better the fit.

^1^ p-value: acceptable ≥ 0.05

^2^ CFI: acceptable ≥ 0.90

^3^ GIF: acceptable ≥ 0.90

^4^ RMSEA: acceptable ≤ 0.08

^5^ chisq/df: acceptable ≤ 3.0

^6^ Lowest AIC and BIC, highest chi-square/df, and highest R^2^ work for nature indicate the best fitting model

## Discussion

### Summary of results

The aim of this paper is to advance the conceptualisation and assessment of human-nature relationships to better understand the premises of children’s desire to protect nature. In summary, the results show that:

- Experiencing the SP enables children to imagine what the life of a salamander is, to feel empathy and concern for them and other animals, and to appreciate the reasoning behind actions of nature conservation.
- Children’s relationship with salamanders changes drastically once they overcome the fear of touching the first salamander (‘yuck’ barrier).
- Children who attend the school responsible for the SP have higher empathy towards salamanders than children who don’t, even before participating in the SP.
- The quantitative methods used to assess children’s psychological and contextual HNC cannot distinguish between a child who participated in the SP and a child who did not.
- Female children show higher values across all indicators of psychological and contextual HNC, and higher desire to work for nature than male children.
- What children mean by *home*, *nature*, and *city* is a collection of meaningful everyday activities, social contexts, and emotions.
- Integrating contextual with psychological factors of HNC improves the prediction of children’s desire to work for nature.
- Self-city and home-city closeness represent a Human Nature Disconnection (HND) that is negatively linked to children’s desire to work for nature.

Overall, the SP seems to be a formative experience for children’s relationship with nature even if the changes in how children value salamanders, animals, and nature in general could not quantified. The SP shares many conditions that characterize the most effective programs in environmental education: occurring over an extended period of time, learning about environmental issues and practising action skills, experiencing and taking ownership of environmental issues, and participating with role models [71]. Accordingly, the interviews suggest that participating in the SP achieves many targets of successful environmental education: ecological knowledge, environmental awareness, practical skills, environmental attitudes and intentions, and enjoyment of the experience [50,72].

Yet, there is a visible discrepancy between the results of the interviews and the assessment of psychological and contextual HNC. Psychological and contextual HNC do not change after children participate in the SP. Regardless of the unexplored and potentially speculative motivations for this discrepancy, recognising such discrepancy is further testimony to the value of interdisciplinary approaches to HNC. Identifying this discrepancy hints that in order to better conceive children’s relationship with nature and understand the premises of their desire to protect nature, HNC has to be operationalised, analysed, and discussed jointly from a multitude of disciplinary perspectives. The aim of this paper is to do exactly so and the results of this study promotes such opportunity.

In the sections below, I combine the findings above to advance future operationalisations and assessments of human-nature relationships. The interdisciplinary findings of this study- with the associated roman numeral in the list above—are discussed in three following sections: conceptualizing human-nature relationships; assessing human-nature relationships, and children’s desire to work for nature and the everyday habitat.

### Conceptualizing human-nature relationships

The interdisciplinary analysis of the results suggests two properties of human-nature relationships that are core characteristics of system thinking [73] and embodied ontologies [74,75]. First, HNC and HND can be interpreted as systems of meaningful relations between mind, body, culture, and environment. Second, they seem to progress non-linearly through complex dynamics with potential delays between causes and effects. In the sections below, I discuss how the findings of this study suggest these two properties.

#### HNC and HND as systems of meaningful relations

Most conceptualisations of HNC commonly suggest a separation between mind, body, and spatial and cultural context [2]. Several findings of this study indicate that this separation is arbitrary and limiting. For instance, this study shows that integrating contextual relations between self, home, nature, and city predict better children’s desire to work for nature than using psychological factors alone (vii). Home-nature and home-city closeness contextualise children’s HNC in the local space and culture. This means that children’s relationship with nature can predict their desire to work for nature better when it is spatially and socially contextualised. This result is supported by many in sense of place literature. Place attachment is a known driver for actions of nature conservation [6,76] and Masterson et al. [77] suggest that nature protective social norms are inherently embedded in a place. Moreover, Kyle at al. [78] state that a sense of connection with a space is also function of the value attributed to the social relations in that space. Children’s HNC can be conceived as a system of relations between themselves and their context.

This study also shows that contextual relations can hamper, rather than promote, children’s desire to work for nature (viii). I aggregated these relations in what I preliminary called *Human-Nature Disconnection* (HND). HND is children’s identification with a system of meanings that demotivate their desire to work for nature. The thematic analysis suggests that those meanings relate to personal closeness to shopping, urban activities, or cars (vi). HND is further indication that human-nature relationships embed spatial and social context. Including the systems of positive and negative contextual meanings predicts children’s desire to work for nature more appropriately (vii).

HNC and HND are conceptually similar to the opposing categories of biophilia and biophobia [79]. Biophilia is an affinity with specific attributes of the natural world that human beings have developed throughout evolution [80]. Conversely, phobic situations involve spiders, snakes, heights, or other attributes that have posed dangers to humans throughout evolution [79]. Like biophilia and biophobia, HNC and HND develop through time—albeit on a shorter time scale than human evolution—shaping appreciation or repulsion for future nature-related experiences. It could be said that HNC and HND are systems of meaningful human-nature relations acquired through personal living that supplement the biophilic and biophobic relations acquired through evolution.

Inseparable interdependencies between self and context in human-nature relationships are also supported by another result of this study (iii). A salamander-friendly culture is the social background of only those children who attend the school responsible for the SP. In this school, children are indirectly and unconsciously exposed to a culture of acceptance and appreciation towards salamanders. This social context coincides with higher levels of empathy towards salamanders even before children participate in the SP (iii). However, participating in the SP does not further increment such empathy (iv). This result should not be considered a failing of the program, nor a failing of the methods used, but an additionally indication that children’s relationship with nature is context dependent and it is not solely identifiable through psychological analysis. These findings suggest that in this situation children’s empathy towards salamanders is more indicative of a social background than of a psychological trait.

Relational and systemic properties of can be also found in the meaning of the very concept of *nature*–as well as in the concepts of *home* and *city*. Children’s meaning of *nature* is a meaningful agglomeration of everyday activities, social contexts, and emotions (vi). Nature, in children’s minds, is not an abstract and universal concept as assumed by psychometric measurements, but a system of meaningful relations that includes physical environments (e.g. plants, animals, and fresh air), emotions (e.g. fun, peace), actions (e.g. play), and culture (e.g. freedom). This personal system of relations is what children connect to and it is what they consider when they reply to psychometric surveys like the CNI and INS used in this study. Implicitly, assessing children’s ‘enjoyment of nature’ (CNI) or closeness to nature (INS) means assessing attachment to a contextualised system of relations that embeds natural spaces and shared social values. Hence, psychological HNC is different when its geographical and cultural context is different. Not only nature experiences are embedded in social and political contexts as Clayton et al. [32] advocate, but this study also suggests that the emerging human-nature relationship is inseparable from its spatial and cultural context.

The last results that support a relational and systemic interpretation of human-nature relationships are those related to gender differences. Across all quantitative methods used, females have higher nature connection, lower disconnection, and are more inclined to work to protect the environment than males (v). These results echo many other studies that find that females have stronger pro-environmental attitudes and behaviours than men [81–86]. Whether for biological or cultural reasons, these results imply strong interdependencies between mind, body, and culture that should be taken into account when conceptualising human-nature relationships. Otto et al. [87] shows that environmental attitudes are also a function of people’s age. They report that environmental attitudes develop during childhood, consolidate during teenage years, and then decline. This means that the same level of psychological HNC has different interpretations given one’s age, which implies once more that environmental attitudes are embodied in one’s body.

In summary, this study suggest that human-nature relationships are better defined as systems of meaningful relations between mind, body, culture, and environment that can promote (HNC) or hamper (HND) sustainable living. Conceptualising human-nature relationship as systems of embodied relations enables such relationships to be categorised and investigated in new ways. For instance, one could distinguish between systems of child-animals relationships, female-forest relationships, or people-biosphere relationships and eventually explore their role to promote or hamper sustainable living. Operationalising human-nature relationships using embodied ontologies [74,88] and system approaches [73] raises new research questions of high value for future sustainable societies. For example, what are the synergies between gender equality and sustainable mindsets? Which spatial configuration of the human habitat best promote children’s relationship with nature? And—noting existing relations between certain cultural products and social preferences and HND [89,90]—which social contexts hamper environmental attitudes and sustainable living? Giusti et al. [34] show that professionals in the field of connecting children to nature explain HNC as a set of abilities that children can learn when given the appropriate spatial and social circumstances. Considering human-nature relationships as systems of meaningful relations between mind, body, culture, and environment would also align academic knowledge with professionals’ wisdom.

#### HNC and HND progresses through complex dynamics

Current literature mostly proposes linear growth for psychological [91] and contextual [6] dimensions of HNC. Conversely, in line with Otto et al. [87] and Vining and Merrick [33], this study suggests that human-nature relationships evolve following complex dynamics with potential delays between causes and effects. For instance, the contributors to children’s desire to work for nature shown in this study are not all positive (viii). The system of relations shaping HNC is in conflict with the system of relations shaping HND when children consider working for nature in the future. That means that human-nature relationships cannot be evaluated as one linear progression from disconnection to connection. At any given point in time a degree of connection and a degree of disconnection co-exist and they promote, or not, sustainable living. Similarly, biophobic attitudes co-exist with biophilic attributes. As this study shows, children’s desire to work for nature in this study is affected not only by the strength of positive relations, but also by the weakness of their negative relations (viii). The progression of HNC and HND and their eventual contribution to sustainable living is therefore dictated by the complex, and conflicting, interactions of their meanings (vi).

Overcoming the ‘yuck barrier’ is another indicator that human-nature relationships follow complex rather than linear dynamics (ii). This experience is similar to what is known as ‘environmental epiphany’ [33]. Before the SP, salamanders are “*…just salamanders*” (Loreta). After the SP, salamanders are considered animals with feelings, pain, and life struggles to which children can relate (i). This is not a linear increment in children’s HNC, but a transformative change in the structure that shape their relationship with nature. After overcoming the ‘yuck barrier’, children rely on new relations between their body (first time holding a salamanders), mind (appreciation rather than disgust), and context (new support from peers). This new system of relations is consolidated in memory [44] and enables them to approach and appreciate a whole new set of nature situations, actions, emotions, and behaviours in the future. This dynamic is a clear example of the embodied progression of children’s HNC proposed by Giusti et al. [34]. Children’s HNC progresses from being comfortable with salamanders (i.e. touching salamanders) to enjoy interact with them (i.e. document species and gender) to being able to care for them (i.e. feeling care and concern). Ultimately, the ‘yuck barrier’ is an indication of non-linear dynamics that further challenges disembodied conceptualisations of HNC and HND.

### Assessing human-nature relationships

The insights from the multi-dimensional and multi-method investigation performed in this study suggest several ways to improve future assessments of human-nature relationships. One salient result is that the impact of participating in the SP on children’s HNC could not be quantified with the tools used here (iv). Despite children saying that they have “*become more nature-friendly*” (Johan) and “*have much more of a sense for nature*” (Megan), the psychometric methods used do not report any statistical difference in how children enjoy, feel empathy, or feel connected to nature. In short, none of these tools could distinguish between a child who participated in the SP from a child who did not (iv). This discrepancy in assessments is a major obstacle for any educational activity that is designed to promote children’s HNC and that requires an evaluation of its effectiveness. Below, I present some limitations of the methods used here and discuss potential ways to improve future assessments of human-nature relationships.

Despite being a child-friendly videogame, *FlexiTwins* has limitations when applied to real-world situations with children. First, the level of attention required to generate accurate results is incompatible with groups of children. In this paper, 30% of the participants’ scores have to be discarded because they are inconsistent or because of high error rates. Second, despite paying a considerable amount of attention to the selection of words used in FlexiTwins, previous research suggests that results might also be influenced by the translation to Swedish, the valence of the words chosen, and the cultural and geographical nuances associated with such words [27,92].

The assessment of children’s psychological HNC allows for further considerations. The group of children in the school responsible for the SP shows significantly higher empathy towards salamanders than the control group, even before participating in the project (iii). Yet, the subscale ‘empathy towards animals’ in the CNI, from which the scale ‘salamander empathy’ is created does not. These results suggest that the generality and de-contextualization of existing psychometric scales impede the identification of changes in psychological HNC. As salamanders are not as intangible as the whole animal kingdom, ‘salamander empathy’ efficiently identifies a pre-existing condition of greater association whereas ‘empathy towards animals’ does not. Children show that what they mean by nature includes emotions (fun, peace, love), actions (playing), and culture (freedom). The abstract, ambiguous, and impersonal concept of nature predominantly used in the literature to assess human-nature relationships [2] might be a strong limiting factor of existing psychometric assessments.

Those limitations are recognised in the literature [42,93]. These methods are often validated within laboratory conditions [4,94], in relation to self-reported environmental behaviours [95], and they can often be considered markers of an overarching construct [91]. Thus, it stands to question if the available psychometric measurements can measure the psychological predictors of environmental actions and sustainable living in real-world situations. There is no clear answer to this question yet. Following the results of this study, the assessment of psychological HNC alone is limited by the operationalisation of abstract and de-contextualised concepts. Considering that human’s existence is dominated by automatic decisional processes [96] and is embedded in space and culture [9], the potential of psychological assessments of human-nature relationships to predict valuable environmental actions and sustainable living might be limited.

These limitations can be addressed in a few ways. First, by taking into consideration the temporal dimension of HNC and HND. Quantitative methods show low accuracy when they assess real-world situations that operate in short time frames [27,28,42]. However, short nature experiences such as the SP might still influence children’s HNC, as suggested by the interviews here (i), but the effects of such changes might be quantifiable only after longer time. Temporal delays between causes and effects are common in system dynamics [73]. Psychometric tools have shown important results when assessing the impact of nature routines [13,34], so they might be more suited for long-term evaluations. Second, exploring which and what kind of spatial and social relations promote or hamper human-nature relationships can be achieved using inductive methodologies. For instance, ethnographic assessments of nature experiences, as in Elliot et al. [28], and qualitative explorations of the system of relational meanings that constitute HNC and HND might offer novel insights in how to develop quantitative and contextualised methods of assessments. Third, human-nature relationships not only increase or decrease, but they combine (as HNC and HND do for children’s desire to work for nature) and transform (as by overcoming the ‘yuck barrier’) in context. As already suggested elsewhere [34], different stages of development for human-nature relationships might exist. This implies that assessing human-nature relationships cannot rely solely on single and linear pre-defined indicators. Multi-methods evaluations should become the norm to assess human-nature relationships. Ultimately, to overcome the limitations listed above assessments should start from the premise of contextualised, embodied, and systemic conceptualisations of human-nature relationships.

### Children’s desire to work for nature and the everyday habitat

From the results of this study is possible to draw some insights about the premises of children’s desire to protect nature and how they can be promoted. Human-nature relationships are often assumed in the literature to be predictors for environmental actions or sustainable living. This is the case whether the predicted outcome is actions of nature conservation [14], sustainable futures [9], or specific pro-environmental behaviours [97–100]. This study contributes to this literature with new insights what promotes or hampers children’s desire to protect the environment. In this study, contextualising children’s relationship with nature in space and culture improves the prediction of their desire to work for nature by about 15% (vii, viii). This prediction comprises the negative influence of children’s closeness to shopping, urban activities, and cars, or indifference to pollution and fumes (vi). This model is, by some margin, the best one to explain the premises of children’s commitment, even if hypothetical, to environmental actions (viii).

These results suggest that the everyday habitat might hamper children’s motivation to protect the biosphere. However, the opposite is also true. Children in the school responsible for the SP show high levels of empathy towards salamanders (iii). This implies that the everyday social and spatial habitat can also promote human-nature relationships favourable to environmental actions or sustainable living. This is also supported by other studies on nature routines [13]. The conscious and unconscious interactions with natural elements occurring in children’s lives form the basis of their nature routines. Nature-rich or nature-poor routines are embedded in children’s everyday habitat and they either promote or hamper children’s desire to protect nature. It follows that if the goal is to understand the predictors of sustainable actions, lifestyles, and cultures more attention should be paid to qualities of the everyday human habitat.

It follows that changing the everyday human habitat should form a central part of any interventions to promote environmental actions and sustainable living. Ensuring a spatial and cultural context suitable for sustainable living is as important as–if not more–individuals’ education. Literature in environmental education recognises this need to shift from indoor individual learning to outdoor community building [101,102]. The spatial human habitat can be designed to be a constant reminder of what a sustainable relationship with nature entails. The literature on biophilic design [103] and nature-connecting habitats [104,105] rests exactly on this conviction. Spatial and cultural interventions on the human habitat have great potential to enable the kind of human-nature relationships required for sustainable living.

## Conclusion

Many in academia suggest that human-nature relationships can be used as a tool to transform human’s unsustainable trends of development [11,12]. The results of this study counter the common assumption that only *one* sustainable human-nature relationship exists and that can be universally measured. De-contextualizing human-nature relationships from culture and space has created an array of useful and valid psychometric measurements that are however limited when applied to real-world situations. The findings of this study emphasize the embodied, contextual, and systemic properties of children’s relationship with nature. The results suggest that human-nature relationships are better defined as systems of meaningful relationships between mind, body, culture, and environment that can promote (i.e. HNC) or hamper (i.e. HND) sustainable living. These systems of relations likely progress through complex dynamics in which the everyday spatial and social habitat plays a major role.

Future operationalisations of HNC and HND would profit from adopting embodied ontologies [74,75] and system thinking [73], as exemplified by the concepts of embodied ecosystems [88] and existing research on children’s HNC [34]. Accordingly, future assessments of children’s relationship with nature would benefit from mixed-methods approaches that explicitly acknowledge the defining role of spatial and cultural contexts. This can be performed through a combination of short-term qualitative and long-term quantitative assessments. Analysing children’s nature routines and their everyday habitat are research efforts of particular promising value to understand the predictors of environmental actions and sustainable living. In conclusion, conceiving and operationalising human-nature relationships as systems of relations would provide novel and valuable insights to promote the psychological and social determinants of resilient sustainable societies.

## Supporting information

S1 Appendix Interview guide. Interview guide used in this study to assess children’s experiential dimension of HNC.
(PDF)

S2 Appendix Booklet used for assessment (English). Translation in English of the booklet used in this study to assess children’s psychological and contextual dimensions of HNC.
(PDF)

S3 Appendix Booklet used for assessment (Swedish). Booklet used in this study to assess children’s psychological and contextual dimensions of HNC.
(PDF)

S1 data Csv data file. Data utilised for the statistical analysis.
(CSV)
