# Supplementary material for: Human-nature relationships in context. Experiential, psychological, and contextual dimensions that shape children’s desire to protect nature
Source: PLoS One. 2019 Dec 5;14(12):e0225951. doi: 10.1371/journal.pone.0225951 (PMC6894778; doi:10.1371/journal.pone.0225951)
Supplement: S1 Appendix — Interview guide used in this study to assess children’s experiential dimension of HNC. (PDF) [file pone.0225951.s001.pdf]

S1 Appendix. Interview guide.

Interview guide used in this study to assess children's experiential dimension of HNC.

- What is the best thing about the Salamander Project for you?
- Is there anything that you think is not good about the project?
- Do you have a special memory or story from the salamander project you could tell me about?
- Could you explain for me how it feels to 'work' with salamanders?
- How does it feel to be part of the project?
- What was different between the first and the last time you participated in the project? (Did something feel different?)
- Can you tell me about what you have learnt from the project?
- Was it different to the way you learn things in the classroom? (How?)
- In what way(s) have your feelings towards salamanders changed with the project? (if they have changed)
- Do you feel like you have changed a bit yourself? (In what way?)
