# Supplementary material for: Human-nature relationships in context. Experiential, psychological, and contextual dimensions that shape children’s desire to protect nature
Source: PLoS One. 2019 Dec 5;14(12):e0225951. doi: 10.1371/journal.pone.0225951 (PMC6894778; doi:10.1371/journal.pone.0225951)

S2 Appendix. Booklet used for assessment (English).

Translation in English of the booklet used in this study to assess children's psychological and contextual dimensions of HNC.

You, Nature, and Home

Name ____________________

Class ____________________

How much do you agree with the following statements? Cross a box

statements	Agree very much	Agree	Neither agree nor disagree	Disagree	Disagree very much
I like to hear different sounds in nature	☐	☐	☐	☐	☐
I like to see wild flowers in nature	☐	☐	☐	☐	☐
When I feel sad, I like to go outside and enjoy nature	☐	☐	☐	☐	☐
I feel calm when I am out in nature	☐	☐	☐	☐	☐
I enjoy working I the garden	☐	☐	☐	☐	☐
Collecting rocks and shells is fun (for example stones and feathers)	☐	☐	☐	☐	☐
I feel sad when wild animals are hurt	☐	☐	☐	☐	☐
I like to see wild animals live in a clean environment	☐	☐	☐	☐	☐
I like to touch animals and plants	☐	☐	☐	☐	☐
I think it's important to take care of animals	☐	☐	☐	☐	☐
People are part of nature	☐	☐	☐	☐	☐
People cannot live without it plants and animals	☐	☐	☐	☐	☐
Being outdoors makes me happy	☐	☐	☐	☐	☐
My actions will affect nature	☐	☐	☐	☐	☐
Picking up trash on the ground can help the environment	☐	☐	☐	☐	☐
People do not have the right to change nature	☐	☐	☐	☐	☐

How much do you agree with the following statements? Cross a box

	Agree very much	Agree	Neither agree nor disagree	Disagree	Disagree very much
I feel sad when salamanders get injured	☐	☐	☐	☐	☐
I like to see salamanders live in a clean environment	☐	☐	☐	☐	☐
I like to touch salamanders	☐	☐	☐	☐	☐
I think it's important to take care of salamanders	☐	☐	☐	☐	☐

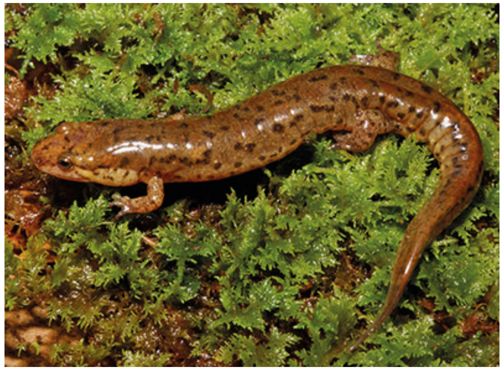

Would you like to work for nature or environmental projects in the futurev?

____

____

Circle the letter that best describes the relationship between home and nature

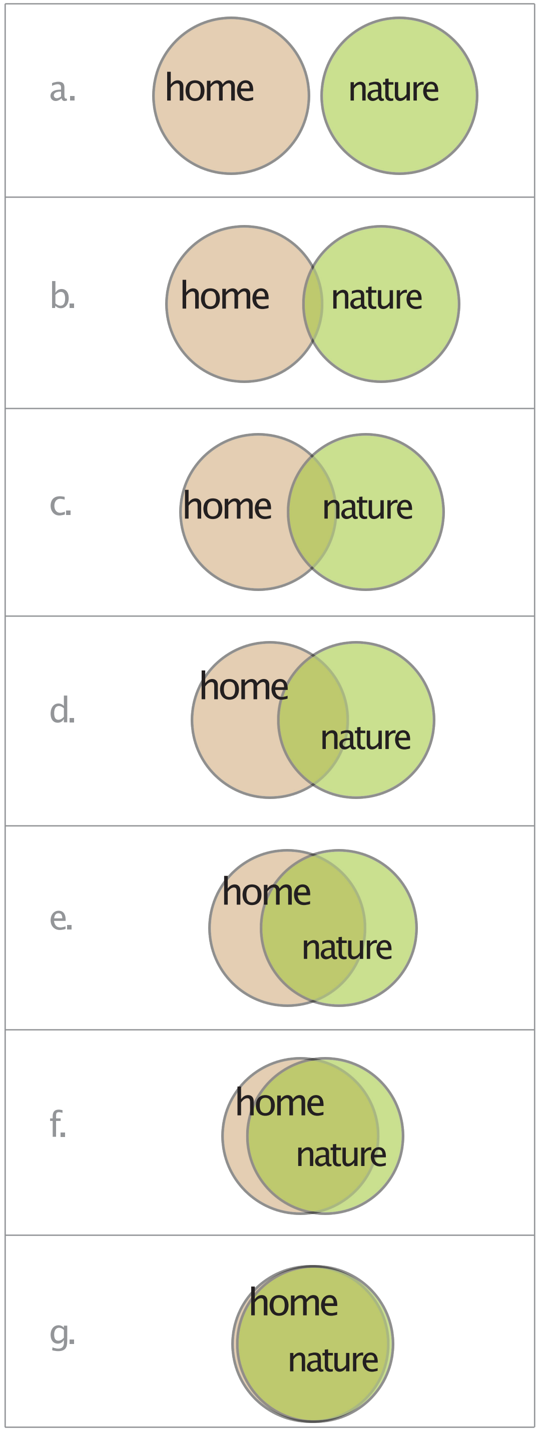

Circle the letter that best describes the relationship between home and city

a. 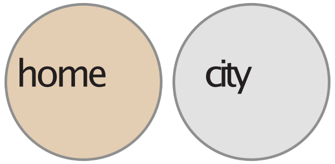

b. 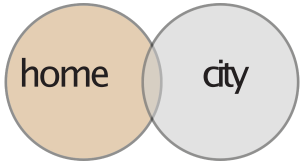

c. 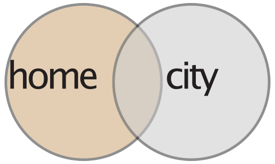

d. 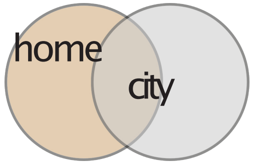

e. 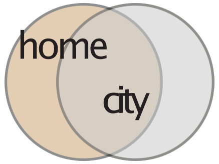

f. 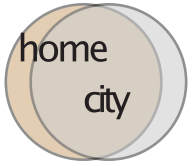

g. 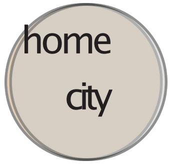

What does the city mean to you ?

____

____

What is best about the city ?

____

____

What is worst about the city?

____

____

What does nature mean to you ?

What is best about nature?

What is worst about nature?

What does home mean to you ?

What is best about home?

What is worst about home?

Circle the letter that best describes the relationship between you and nature

a. 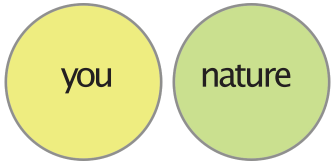

b. 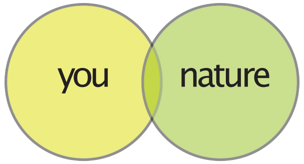

c. 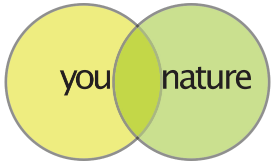

d. 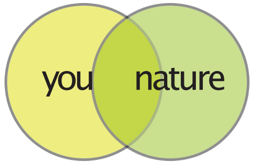

e. 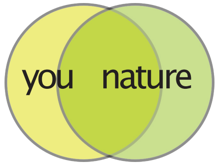

f. 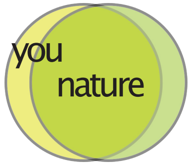

g. 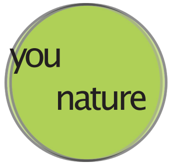

Circle the letter that best describes the relationship between you and city

a. 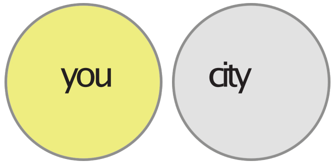

b. 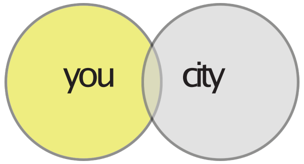

c. 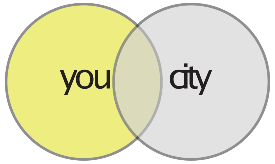

d. 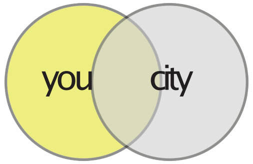

e. 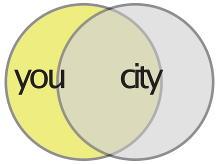

f. 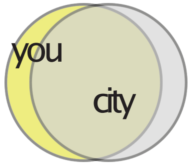

g. 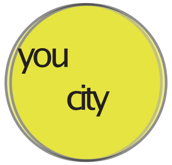

Supplement: S2 Appendix — Translation in English of the booklet used in this study to assess children’s psychological and contextual dimensions of HNC. (PDF) [file pone.0225951.s002.pdf]
