# Supplementary material for: Human-nature relationships in context. Experiential, psychological, and contextual dimensions that shape children’s desire to protect nature
Source: PLoS One. 2019 Dec 5;14(12):e0225951. doi: 10.1371/journal.pone.0225951 (PMC6894778; doi:10.1371/journal.pone.0225951)

S3 Appendix. Booklet used for assessment (Swedish).

Booklet used in this study to assess children's psychological and contextual dimensions of HNC.

Du, Naturen, och Hemma

Namn ______________________________

Klass ______________________________

Hur mycket håller du med om följande påståenden?
Kryssa en ruta

Påståenden	Stämmer mycket bra	Stämmer	Håller varken med eller inte	Stämmer inte	Stämmer inte alls
Jag tycker om att höra olika ljud i naturen	☐	☐	☐	☐	☐
Jag tycker om att se vilda blommor i naturen	☐	☐	☐	☐	☐
När jag är ledsen tycker jag om att vara ute och leka i naturen	☐	☐	☐	☐	☐
Jag känner mig lugn när jag är ute i naturen	☐	☐	☐	☐	☐
Jag tycker om att arbeta I trädgarden	☐	☐	☐	☐	☐
Att samla på saker från naturen är kul (t.ex. stenar, fjädrar)	☐	☐	☐	☐	☐
Jag känner mig ledsen när vilda djur blir skadade	☐	☐	☐	☐	☐
Jag tycker om att se vilda djur leva i en ren miljö	☐	☐	☐	☐	☐
Jag tycker om att röra vid djur och växter	☐	☐	☐	☐	☐
Jag tycker det är viktigt att ta hand om djur	☐	☐	☐	☐	☐
Människor är en del av naturen	☐	☐	☐	☐	☐
Människor kan inte leva utan växter och djur	☐	☐	☐	☐	☐
Att vara utomhus gör mig glad	☐	☐	☐	☐	☐
Mina handlingar kommer att påverka naturen	☐	☐	☐	☐	☐
Att plocka upp skräp från marken kan hjälpa miljön	☐	☐	☐	☐	☐
Folk har inte rätt att ändra på naturen	☐	☐	☐	☐	☐

Hur mycket håller du med om följande påståenden?
Kryssa en ruta

Påståenden	Stämmer mycket bra	Stämmer	Håller varken med eller inte	Stämmer inte	Stämmer inte alls
Jag känner mig ledsen när salamandrar blir skadade	☐	☐	☐	☐	☐
Jag tycker om att se salamandrar leva i en ren miljö	☐	☐	☐	☐	☐
Jag tycker om att röra vid salamandrar	☐	☐	☐	☐	☐
Jag tycker det ar viktigt att ta hand om salamandrar	☐	☐	☐	☐	☐

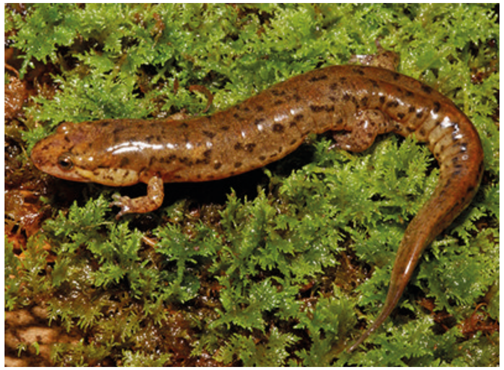

Skulle du kunna tänka dig att arbeta med något natur–eller miljöprojekt i framtiden?

____

____

Ringa in bokstaven som bäst beskriver relationen mellan hemmet och naturen

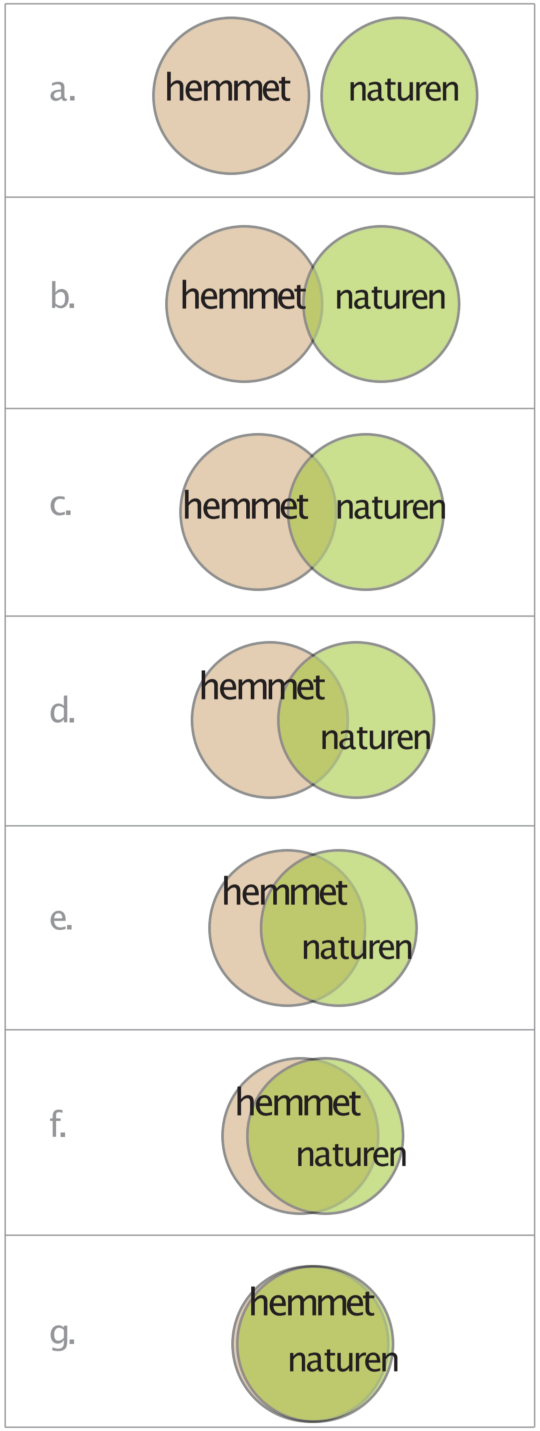

Ringa in bokstaven som bäst beskriver relationen mellan hemmet och staden

a. 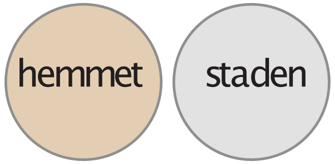

b. 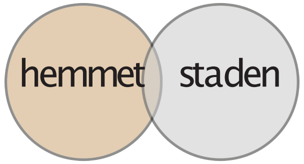

c. 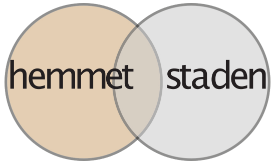

d. 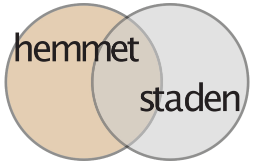

e. 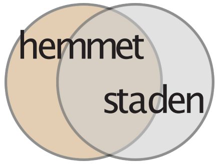

f. 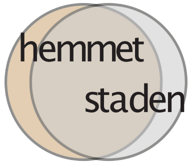

g. 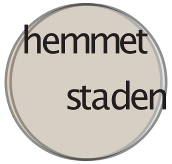

Vad betyder staden för dig?

____

____

Vad är det bästa med staden ?

____

____

Vad är det värsta med staden ?

____

____

Vad betyder naturen för dig?

Vad är det bästa med naturen?

Vad är det värsta med naturen?

Vad betyder hemmet för dig?

Vad är det bästa med hemmet?

Vad är det värsta med hemmet?

Ringa in bokstaven som bäst beskriver relationen mellan dig och naturen

a.

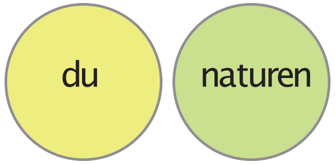

b.

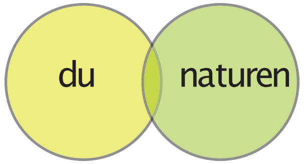

c.

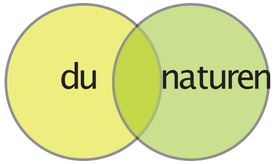

d.

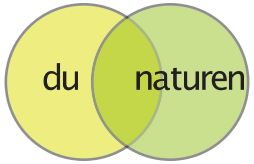

e.

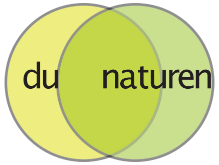

f.

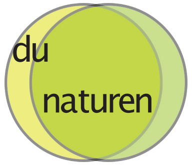

g.

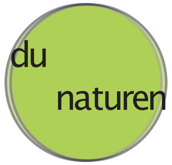

Ringa in bokstaven som bäst beskriver relationen mellan dig och staden

a.

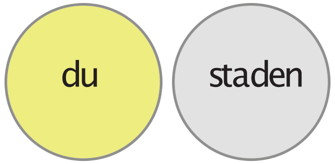

b.

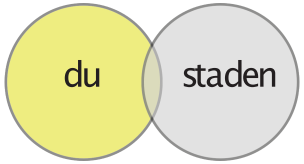

c.

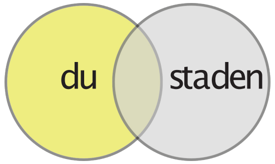

d.

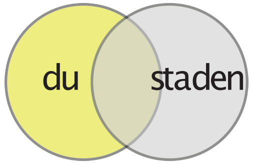

e.

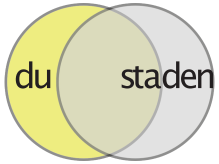

f.

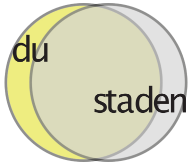

g.

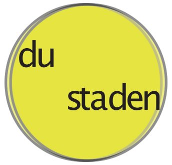

Supplement: S3 Appendix — Booklet used in this study to assess children’s psychological and contextual dimensions of HNC. (PDF) [file pone.0225951.s003.pdf]
